# A single-nucleotide polymorphism in *MMP9* is associated with decreased risk of steroid-induced osteonecrosis of the femoral head

**DOI:** 10.18632/oncotarget.12034

**Published:** 2016-09-15

**Authors:** Jieli Du, Wanlin Liu, Tianbo Jin, Zhenqun Zhao, Rui Bai, Huiqin Xue, Junyu Chen, Mingqi Sun, Xiyang Zhang, Guoqiang Wang, Jianzhong Wang

**Affiliations:** ^1^ Inner Mongolia Medical University, Hohhot, Inner Mongolia, 010050, China; ^2^ Department of orthopedics and Traumatology, The 2nd Affiliated Hospital of Inner Mongolia University, Hohhot, Inner Mongolia, 010030, China; ^3^ The College of Life Sciences, Northwest University, 710069, China

**Keywords:** MMP9, MMP2, single nucleotide polymorphisms, osteonecrosis of the femoral head, association study

## Abstract

Osteonecrosis of the femoral head (ONFH) is a common hip joint disease, and steroid-induced ONFH accounts for a large number of cases. Here, we examined eight previously-identified single-nucleotide polymorphisms (SNPs) in the *MPP2* and *MPP9* genes of 285 steroid-induced ONFH patients and 507 healthy controls from northern China to determine whether these SNPs were associated with the risk of developing steroid-induced ONFH. Chi-squared tests and genetic model and haplotype analyses were used to evaluate associations. The rs2274755 SNP in *MMP9* was associated with a decreased risk of steroid-induced ONFH in the allele, dominant, and additive models. Additionally, the “CGC” *MMP9* haplotype was associated with a 0.69-fold decrease in the risk of steroid-induced ONFH. Although additional, larger population-based studies are needed to confirm these findings, our results reveal for the first time an association between a *MMP9* SNP at the rs2274755 locus and a decreased risk of steroid-induced ONFH in a northern Chinese population.

## INTRODUCTION

Osteonecrosis of the femoral head (ONFH) is a common hip joint disease that frequently causes disability. Nearly 20 million patients suffer from ONFH worldwide [1], with an estimated 8.12 million non-traumatic ONFH cases in 2013 in China alone [2]. Steroid-induced ONFH accounts for 24.1% of all ONFH cases [1]. Several studies demonstrate that only some patients develop ONFH within a few months of glucocorticoid (GC) therapys, suggesting that genetic factors may confer susceptibility or resistance to steroid-induced ONFH.

Matrix metalloproteinases (MMPs) are a family of zinc-dependent proteolytic enzymes that participate in morphogenesis, wound healing, tissue repair, and remodeling in response to injury, and glucocorticoids can inhibit the expression of MMPs. Garvican *et al*. [3] found that corticosteroids suppressed the expression of MMP-1, MMP-3, MMP-13, gelatinase A (MMP-2), and gelatinase B (MMP-9) mRNA. Glucocorticoids also down-regulate MMP-2 expression by inducing microRNA 29c [4]. Dexamethasone might aid in the treatment of bacterial meningitis and acute lung injury by down-regulating MMP-9 expression [5, 6]. In addition, gelatinase A (MMP- 2) and B (MMP-9) play crucial roles in bone remodeling [7]. Both gelatinase A (MMP-2) and gelatinase B (MMP-9) are produced by osteoblasts, and MMP-9 can also be synthesized by osteoclasts [8]. MMP-2 expression is upregulated in osteoarthritic cartilage [9], and MMP-9, which regulates growth plate angiogenesis and apoptosis of hypertrophic chondrocytes, may be essential for normal bone remodeling [10]. Both MMP-2 and MMP- 9 expression are increased in the joints of rheumatoid arthritis patients [11, 12]. Additionally, MMP-2 mRNA and protein levels are up-regulated in ONFH subjects, while MMP-9 protein is overproduced in osteoarthritic bone tissue [8]. Inactivating mutations in the *MMP2* gene result in bone and joint features of multicentric osteolysis with arthritis [13], and osteoclast MMP-9 expression increases in response to adrenocorticotropic hormone (ACTH) in murine models, confirming the effects of ACTH on early osteoclast differentiation [14]. Furthermore, observations that low ACTH levels are associated with microvascular osteonecrosis and that ACTH administration reduces this necrosis suggest that ACTH might be useful for decreasing the risk of osteonecrosis in humans [14]. Additionally, MMP-9 expression may serve as a marker of efficacy in the treatment of steroid-induced osteonecrosis using ACTH. MMP-2 and MMP-9 expression, which were both higher in steroid-induced ONFH patients than in controls, have only recently been investigated in Chinese populations.

Genetic polymorphisms are normal genetic variations found in more than 1% of the population that can influence protein transcription and the expression of related genes to contribute to an individual's disease susceptibility [15]. *MMP2* and *MMP9* single nucleotide polymorphisms (SNPs) have been associated with several diseases. For example, rs1053605 and rs243849 polymorphisms are associated with stroke outcome [16], but not with obesity [17], thoracic aortic dissection [18], or endometriosis [19]. rs243847 polymorphism might increase the risk of non-vertebral osteoporotic fractures [20] and intracranial aneurysms in male patients [21]. rs243832 and rs7201 polymorphisms are associated with an increased risk of endometriosis [19]. rs3918249 polymorphism is associated with a decreased risk of glaucoma, especially in Caucasian patients [22], and an increased risk of childhood-onset asthma [23]; in the same study, rs2274755 polymorphism was also associated with an increased risk of asthma. Gao *et al*. [24] suggested that the rs3918254 locus may be susceptible to primary angle-closure glaucoma in Chinese patients. However, associations between these eight SNPs and steroid-induced ONFH have not yet been examined. The aim of this study was to determine whether s(SNPs in the *MMP2* and *MMP9* genes were associated with the risk of steroid-induced ONFH in a northern Chinese patient population.

## RESULTS

Sex and age distributions for ONFH patients and controls are shown in Table 1. 285 ONFH patients (112 female, 173 male) and 507 controls (111 female, 396 male) were recruited for the present study. The mean ages were 41.88±12.79 years for patients and 47.43 ± 9.74 years for controls.

**Table 1:** Characteristics of cases and controls in this study

Variable(s)	Case (*n* = 285)	Control (*n* = 308)	*p* value
Sex N(%)			< 0.001^a^
Male	173 (60.7)	396 (78.1)	
Female	112(39.3)	111 (21.9)	
Age, year (mean ± SD)	41.88 ± 12.79	47.43 ± 9.74	< 0.001^b^

a Two-sided Chi-squared test.

b Independent samples *t* test.

Eight SNPs in *MMP2* and *MMP9* were analyzed in this study. Allele frequencies and basic information for all SNPs are shown in Table 2. All eight SNPs exhibited Hardy–Weinberg equilibrium in control subjects (*p* > 0.05), while rs2274755 was associated with a decreased risk of steroid-induced ONFH (OR = 0.70, 95% CI: 0.51–0.96, *p* = 0.025).

**Table 2:** Allele frequencies in cases and controls and odds ratio estimates for steroid-induced ONFH

SNP ID	Gene	Position	Alleles A/B	MAF		*p*^a^ value for HWE	ORs	95% CI		*p*^b^
				case	control					
rs1053605	MMP2	16q12.2	T/C	0.11	0.15	0.843	0.75	0.54	1.04	0.085
rs243849	MMP2	16q12.2	T/C	0.21	0.20	1.000	1.03	0.78	1.35	0.858
rs243847	MMP2	16q12.2	C/T	0.82	0.68	0.117	1.20	0.97	1.47	0.090
rs243832	MMP2	16q12.2	C/G	0.57	0.62	0.347	0.92	0.74	1.13	0.424
rs7201	MMP2	16q12.2	C/A	0.37	0.34	0.349	1.07	0.85	1.36	0.551
rs3918249	MMP9	20q13.12	T/C	0.44	0.48	0.613	0.93	0.75	1.16	0.525
rs2274755	MMP9	20q13.12	T/G	0.12	0.18	0.729	0.70	0.51	0.96	0.025^*^
rs3918254	MMP9	20q13.12	T/C	0.28	0.23	0.664	1.19	0.93	1.54	0.173

SNP single nucleotide polymorphism, HWE Hardy-Weinberg equilibrium, OR odds ratio, 95% CI 95% confidence interval, MAF minor allele frequency.

* *p* ≤ 0.05.

Bonferroni's multiple adjustment was applied to the level of significance, which was set at *p* ≤ 0.00104 (0.05/48).

a *p* was calculated by exact test.

b *p* was calculated by Pearson Chi-squared test.

We then examined whether harboring the minor allele for each SNP compared to the wild-type allele represented a risk factor in the genetic models shown in Table 3. rs2274755 polymorphism in the *MMP9* gene conferred a protective effect against steroid-induced ONFH in the dominant model both before (OR = 0.66, 95% CI: 0.47–0.94, *p* = 0.019) and after adjustment for age and gender (OR = 0.68, 95% CI: 0.47–0.98, *p* = 0.034), and in the additive model both before (OR = 0.70, 95% CI: 0.51–0.96, *p* = 0.022) and after adjustment for age and gender (OR = 0.70, 95% CI: 0.51–0.98, *p* = 0.033). However, in the over-dominant model, rs2274755 polymorphism was only associated with a decreased risk of steroid-induced ONFH before adjustment for age and gender (OR = 0.67, 95% CI: 0.47–0.96, *p* = 0.026).

**Table 3:** Genotypic model analysis of relationship between SNPs and steroid-induced ONFH risk

Model	Genotype	Group = control	Group = Hormone	Without Adjustment		With Adjustment	
				OR (95% CI)	*p*^a^-value	OR (95% CI)	*p*^a^-value
Codominant	G/G	364 (71.8%)	226 (79.3%)	1		1	
	G/T	133 (26.2%)	55 (19.3%)	0.67 (0.47–0.95)	0.063	0.69 (0.48–1.00)	0.100
	T/T	10 (2%)	4 (1.4%)	0.64 (0.20–2.08)		0.56 (0.17–1.92)	
Dominant	G/G	364 (71.8%)	226 (79.3%)	1		1	
					0.019*		0.034*
	G/T-T/T	143 (28.2%)	59 (20.7%)	0.66 (0.47–0.94)		0.68 (0.47–0.98)	
Recessive	G/G-G/T	497 (98%)	281 (98.6%)	1		1	
					0.550		0.420
	T/T	10 (2%)	4 (1.4%)	0.71 (0.22–2.28)		0.61 (0.18–2.08)	
Overdominant	G/G-T/T	374 (73.8%)	230 (80.7%)	1		1	
					0.026*		0.055
	G/T	133 (26.2%)	55 (19.3%)	0.67 (0.47–0.96)		0.70 (0.48–1.01)	
Log-additive	—	—	—	0.70 (0.51–0.96)	0.022*	0.70 (0.51–0.98)	0.033*

* *p* ≤ 0.05.

Bonferroni's multiple adjustment was applied to the level of significance, which was set at *p* ≤ 0.00104 (0.05/48).

*p* values were calculated by Wald test by unconditional logistic regression adjusted for age and gender.

**Figure 1:**
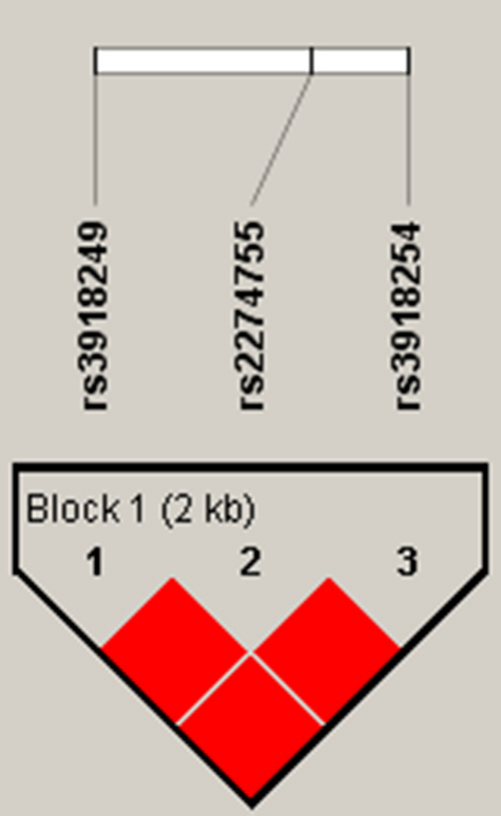
Linkage disequilibrium (LD) plots containing three SNPs from *MMP9* Red squares display statistically significant associations between a pair of SNPs, as measured by D'; darker shades of red indicate higher D'

**Figure 2:**
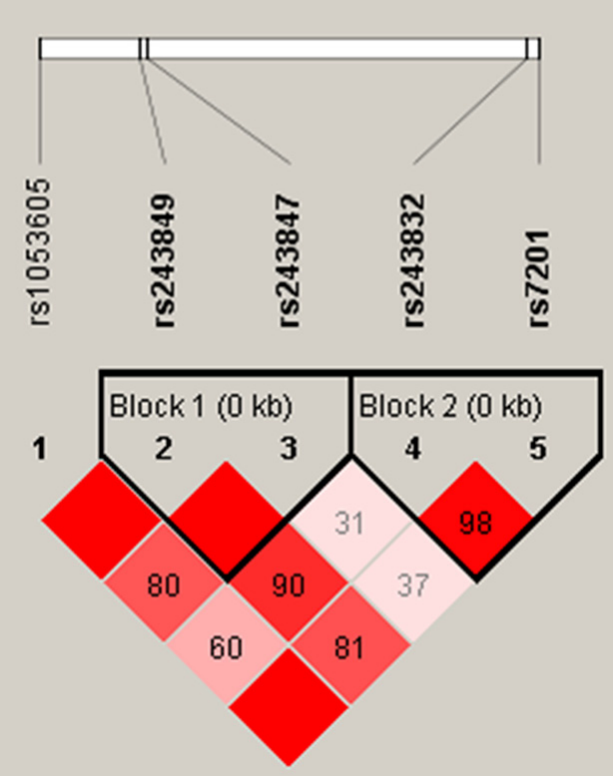
Linkage disequilibrium (LD) plots containing five SNPs from *MMP2* Red squares display statistically significant associations between a pair of SNPs, as measured by D'; darker shades of red indicate higher D'.

Finally, haplotype analysis detected three blocks in the *MMP2* and *MMP9* genes (Figures 1 and 2). rs243849 and rs243847, rs243832 and rs7201, and rs3918249, rs2274755, and rs3918254 had very strong linkage disequilibria; compared to the “CGC” wild-type, the “CTC” haplotype was associated with a decreased risk of steroid-induced ONFH (OR = 0.69, 95% CI: 0.48–0.98, *p* = 0.039) (Table 4).

**Table 4:** The haplotype frequencies of *MMP9* polymorphisms and their association with steroid-induced ONFH risk

	Haplotype				Without Adjustment		With Adjustment	
	rs3918249	rs2274755	rs3918254	Freq	OR (95% CI)	*p*^a^	OR (95% CI)	*p*^b^
1	C	G	C	0.3491	1	—	1	—
2	T	G	C	0.3169	0.88 (0.68–1.13)	0.310	0.91 (0.70–1.18)	0.470
3	C	G	T	0.1976	1.06 (0.80–1.42)	0.680	1.03 (0.76–1.40)	0.850
4	C	T	C	0.1364	0.67 (0.48–0.95)	0.026*	0.69 (0.48–0.98)	0.039*

a *p* value were calculated by unconditional logistic regression.

b *p* values were calculated by unconditional logistic regression adjusted for age and gender.

* *p* ≤ 0.05 indicates statistical significance.

However, after Bonferroni correction was applied to our data, there were no statistically significant associations between *MMP2* and *MMP9* SNPs and the risk of steroid-induced ONFH.

## DISCUSSION

Genetic studies have provided insight into numerous diseases, including osteonecrosis. In this study, eight SNPs in the *MMP2* and *MMP9* genes that have previously been investigated in other diseases were examined in 792 subjects (285 patients with steroid-induced ONFH and 507 healthy controls) to determine whether they were associated with the risk of steroid-induced ONFH in a northern Chinese population. Our results suggest that rs2274755 *MMP9* genetic polymorphism was associated with a decreased risk of steroid-induced ONFH. In addition, the “CGC” *MMP9* haplotype was associated with a 0.69-fold decrease in the risk of steroid-induced ONFH.

MMP-9, an extracellular matrix (ECM)-metabolizing enzyme, plays a crucial role in bone remodeling. Overexpression of MMP-9 by osteoclasts is critical for digesting type I collagen and aggrecan, which are found mainly in cartilage [11]. MMP-9 expression is stimulated by RANKL, which is essential for osteoclastogenesis [25–27]. Fujisaki *et al*. [28] reported that RANKL induced MMP-9 production in osteoclasts and increased bone resorption in the presence of IL-1α. In addition, Guo *et al*. [29] showed that decreased membrane-type matrixmetalloproteinases-1 (MT1-MMP) expression is involved in RANKL signaling in osteoblasts and the activation of osteoclasts. Thus, osteoclast-derived MMP-9 may contribute to bone resorption in steroid-induced ONFH. MMP-9 also facilitates the recruitment of inflammatory cells in the airways during allergic inflammation and chronic obstructive pulmonary disease [30, 31]. Therefore, MMP9 might also contribute to steroid-induced ONFH by affecting bone resorption or inflammatory responses. Additional studies are needed to characterize the exact mechanisms by which MMP-9 regulates steroid-induced ONFH.

The rs2274755 locus is located in an intron (boundary) of the *MMP9* gene, and rs2274755 variations have been investigated in many studies. In a study of a Chinese Han population, no association was found between rs2274755 and susceptibility to either polypoidal choroidal vasculopathy or age-related macular degeneration [32]. Jimenez-Morales *et al*. found that rs2274755 polymorphism was associated with asthma in Mexican pediatric patients [23], as was the case in Nakashima *et al*.'s study in a Japanese population [33]. Furthermore, a functional study suggested that MMP-9 polymorphisms were associated with increased asthma pathogenesis [33]. Here, we found that rs2274755 polymorphism was associated with a decreased risk of steroid-induced ONFH in a northern Chinese population. Thus, rs2274755 variation may have a complex effect on inflammatory cells and capillaries in the femoral head, which may promote steroid-induced ONFH.

To our knowledge, the present study provides the first evidence that variation at the rs2274755 locus in the *MMP9* gene is associated with a decreased risk of steroid-induced ONFH risk in the northern Chinese population. However, some limitations of this study should be considered when interpreting these results. First, the sample sizes were relative small, and additional studies should confirm these results in more patients. Second, all samples were collected at the Zhengzhou Traditional Chinese Medicine Traumatology Hospital in northern China, which may increase the type I error (false positive) rate for association studies. Additionally, the Bonferroni correction for multiple tests is important to account for false discovery rate. After correction, we found no statistically significant associations between *MMP2* and *MMP9* SNPs and the risk of steroid-induced ONFH. This may be due to our small sample size and strict SNP filtering criteria; furthermore, the Bonferroni correction is very conservative when applied to multiple tests. Continued sample collection will help to confirm our results in future studies.

Taken together, our results identified for the first time an association between rs2274755 MMP9 polymorphism and a decreased risk of steroid-induced ONFH in a northern Chinese population. Additional larger population-based studies are needed to confirm these results.

## MATERIALS AND METHODS

### Ethics statement

This study adhered to the principles of the Declaration of Helsinki, and all protocols involving human specimens were approved by the Ethical Committee of the Zhengzhou Traditional Chinese Medicine Traumatology Hospital. Informed consent was obtained from each subject.

### Study participants

285 steroid-induced ONFH patients were enrolled between September 2014 and January 2016 at the Zhengzhou Traditional Chinese Medicine Traumatology Hospital. Steroid-induced ONFH was defined by a history of a mean daily steroid dose of ≥ 16.6 mg, or a high-dose steroid impulsion therapy lasting more than 1 week [34–36]. ONFH was diagnosed by examining osteonecrosis in anteroposterior and frog view X-rays of both hips and/or magnetic resonance imaging [37]. Exclusion criteria were as follows: (i) those who drank the equivalent of more than 400 mL of pure ethanol per week; (ii) those did not meet the diagnostic criteria for steroid-induced ONFH and patients with traumatic ONFH, dislocation of the hip joint, and other hip diseases. (iii) those who had significant familial hereditary diseases.

None of the 507 control subjects recruited based on medical examinations at the Zhengzhou Traditional Chinese Medicine Traumatology Hospital were related to the case subjects. All subjects were Han Chinese from northern China. Subjects with excessive consumption of alcohol, corticosteroids, or significant familial hereditary diseases were excluded.

### SNP genotyping

All eight SNPs had minor allele frequencies > 5% in the HapMap Chinese Han Beijing (CHB) population. Blood samples were collected in EDTA tubes and stored at −80°C after centrifugation at 2000 rpm for 10 minutes. Genomic DNA was extracted from whole blood using an extraction kit (GoldMag, China) and stored at −20°C. DNA quantity was evaluated by spectrophotometry (DU530UV/VIS spectrophotometer, Beckman Instruments, Fullerton, CA, USA). The Multiplexed SNP Mass EXTEND assay was developed using Sequenom MassARRAY Assay Design 3.0 Software. The primers for the eight selected SNPs are shown in Table 5. A Sequenom MassARRAY RS1000 was used for SNP genotyping according to the manufacturer's protocol.

**Table 5:** Primers used for this study

SNP_ID	1st-PCRP	2nd-PCRP	UEP_SEQ
rs1053605	ACGTTGGATGCTCAAA GTTGTAGGTGGTGG	ACGTTGGATGAAGGAGTA CAACAGCTGCAC	AACAGCTGCACTGATAC
rs243849	ACGTTGGATGTACCTTG GTCAGGGCAGAAG	ACGTTGGATGAGTGACGG AAAGATGTGGTG	ACAGCCAACTACGATGA
rs243847	ACGTTGGATGAAGACAA GAGCAGTGACCCC	ACGTTGGATGCCAAAATC AGACCCTGGTAG	ccTGCTGCTACTCACCTCC
rs243832	ACGTTGGATGCCTATGCC AGGCAGAAATTC	ACGTTGGATGGAGAAAGA AGAGACCGTGAC	ACATTCTGGCACACAGAAG
rs7201	ACGTTGGATGTCCAATCC CACCAACCCTCA	ACGTTGGATGGCAGGGCTG CGTTGAAAATA	aAGGGCTGCGTTGAAAATATCAAAG
rs3918249	ACGTTGGATGAAGCACT GGTGTCTGGAAAG	ACGTTGGATGGATTACAAG TGTGAGCCGTC	gaaGTCATGCCCAGCAGGGACTA
rs2274755	ACGTTGGATGGGGAGAG AATGAAGGGAATC	ACGTTGGATGTTCGACGAT GACGAGTTGTG	gCTGGGCAAGGGCGTCGGT
rs3918254	ACGTTGGATGTCTTCGG CTTCTGCCCGAC	ACGTTGGATGCAATACATG ATGAGAGGGCG	CTGGTAGACAGGGTGGA

### Statistical analyses

All statistical analyses were performed using SPSS 19.0 software for Windows (SPSS, Chicago, IL). Allele and genotype frequencies were determined using direct counts. Hardy–Weinberg equilibrium for each SNP was determined using an exact test to compare the expected frequencies of genotypes in controls. All *p* values were two-sided, and *p* ≤ 0.05 was considered statistically significant. Allele and genotype frequencies in ONFH patients and controls were calculated using Chi-squared test/Fisher's exact tests. Associations between SNPs and risk of steroid-induced ONFH were tested in genetic models using SNP Stats software. Odds ratios (ORs) and 95% confidence intervals (CIs) were calculated using unconditional logistic regression analysis with adjustment for gender and age. Finally, the Haploview software package (version 4.2) and SHEsis software platform (http://www.nhgg.org/analysis/) were used to estimate pairwise linkage disequilibrium (LD), haplotype construction, and genetic association at polymorphism loci.
